# Genotype and cardiac outcome in patients with cardiocutaneous syndrome (Naxos disease variant: Carvajal syndrome)

**DOI:** 10.1186/s13023-025-03612-8

**Published:** 2025-03-19

**Authors:** Maha Binfadel, Mohamed Umair Aleem, Mohammed Alhabdan, Nadiah Alruwaili, Zuhair AlHassnan, Olga Vriz, Sahar Tulbah, Dimpna Calila Albert-Brotons

**Affiliations:** 1 Department of Pediatric Cardiology, Heart Center, King Faisal Specialist Hospital & Research Center, 11211 Riyadh, Saudi Arabia; 2 College of Medicine, Alfaisal University, 11533 Riyadh, Saudi Arabia; 3 Clinical Research Department, Heart Center, King Faisal Specialist Hospital & Research Center, 11211 Riyadh, Saudi Arabia; 4 Genetic Department, Faisal Specialist Hospital & Research Center, 11211 Riyadh, King Saudi Arabia; 5 Department of Cardiology, Azienda Sanitaria Universitaria Friuli Centrale, 33100 San Daniele, Udiene, Italy; 6 Department of Translational Genomics, Center for Genomic Medicine, King Faisal Specialist Hospital & Research Center, 11211 Riyadh, Saudi Arabia

**Keywords:** Naxos disease variant, Carvajal syndrome, Woolly hair, Palmoplantar keratosis

## Abstract

**Background:**

Naxos disease variant (Carvajal syndrome) is a cardiocutaneous genetic disease caused by Plakoglobin and Desmoplakin gene mutation, and usually manifests with woolly hair, palmoplantar keratoderma, and cardiomyopathy, and are found to have a high risk for uncontrolled arrhythmia.

**Methods:**

An observational retrospective cohort study was conducted at King Faisal Specialist Hospital & Research Center in Riyadh, Saudi Arabia, a tertiary care hospital, which included 10 Saudi pediatric patients with clinical manifestations that indicate Naxos disease variant. The medical records of the patients were analyzed such as Echocardiography parameters (for ventricular function assessment), electrocardiography (ECG), 24-h Holter (for arrhythmias), and genetic analysis results were collected to confirm the medical diagnosis.

**Result:**

We report 10 Saudi pediatric patients with Naxos disease variant who presented with severe dilated cardiomyopathy manifestation. All the patients had woolly hair, and half had palmoplantar keratoderma. They all had severely dilated and depressed left ventricular systolic function, and nine of them also had depressed right ventricular systolic function. Frequent premature ventricular tachycardias (PVCs) were reported in nine cases, and an implantable cardioverter defibrillator (ICD) was implanted in 3 patients for uncontrolled ventricular tachycardias. Moreover, four patients underwent heart transplantation, and three died suddenly while waiting for a heart donation. Finally, in 8 patients, genetic studies were homozygous for Desmoplakin gene *(DSP)*, confirming the diagnosis.

**Conclusion:**

Naxos disease variant is accompanied by high risk of arrhythmia and sudden cardiac deaths, so family members of proband need an extensive genetic workup for identification of gene carriers for counseling, especially in our Arab countries where consanguineous marriage is common. Moreover, hair and skin phenotypes in a child should alert for signs of cardiomyopathy manifestation.

## Introduction

Naxos disease variant is a rare autosomal recessive genodermatosis first reported in the families descended from the Hellenic Island of Naxos in Greece [1, 2]. Mutations in the genes encoding Desmosomal proteins (Plakoglobin and Desmoplakin) have been determined as the causes of Naxos and Naxos disease variants (MIM# 601,214) [3]. Certain Aegean Islands, Turkey, Israel, and Saudi Arabia have been found to have affected families [3–5]. Families from India and Ecuador had been reported to have a syndrome with a similar cutaneous phenotype with primarily left ventricular involvement (Carvajal syndrome, dilated cardiomyopathy with wooly hair and keratoderma; MIM#605,676) [3, 6, 7].

A key phenotypic difference between Naxos disease and Naxos disease variant lies in their cardiac manifestations. Naxos disease is characterized by arrhythmogenic right ventricular cardiomyopathy/dysplasia (ARVC/D), woolly hair, and palmoplantar keratoderma [3, 8]. Woolly hair is present from birth, while erythema and hyperkeratotic lesions develop later in infancy at pressure points on the hands and feet [3]. Heart disease usually does not manifest in Naxos patients until they reach adolescent age [3]. In contrast, Naxos disease variant (Carvajal syndrome) manifests with cardiomyopathy symptoms, such as left ventricular dysfunction and heart failure, in early childhood [3, 6, 7].

Endomyocardial biopsy performed upon clinical indications reveals myocyte degeneration with strands of surviving myocytes surrounded by fibrous tissue embedded within adipocytes [3]. In 2004, Stuhrmann et al. [9] reported that a 2-year-old girl and her 30-year-old aunt from a Saudi Arabian family were not impacted by the same mutation that was found in the Greek families, which is Pk2157del2 frameshift, concluding that another variant of the gene can also cause Naxos disease variant which was affecting the Saudi Arabian Family in the discussion.

The objective of this study is to describe Saudi pediatric patients affected with woolly hair, cutaneous abnormalities, and abnormal cardiac function who were referred to follow up in the pediatric heart failure clinic at King Faisal Specialist Hospital & Research Center (KFSH&RC), Riyadh, Saudi Arabia, to determine the accuracy of clinical diagnostic criteria and genetics involved in children of Saudi Arabia with suspicion of Naxos disease variant.

## Materials and methods

This study is an observational retrospective cohort analysis of children referred from their local hospitals to the advanced Pediatric Heart Failure Clinic at King Faisal Specialist Hospital & Research Center, Riyadh, Saudi Arabia, between August 2010 and June 2020. All patients presented with dilated cardiomyopathy characterized by significantly depressed left ventricular systolic function and underwent evaluation for heart transplant candidacy.

A distinctive phenotype, including woolly hair and mucocutaneous abnormalities indicative of a Naxos disease variant, was observed in the cohort. Genetic analysis, performed as part of the routine work-up for dilated cardiomyopathy, revealed that eight patients were homozygous for a DSP gene mutation.

### Study population

Our study population includes a total of 10 pediatric patients with age < 14 years at the time of first presentation; criteria of pediatric age in Saudi Arabia. These patients were referred from the local primary care clinics to our pediatric heart failure clinic of a tertiary care hospital. These patients demonstrated cardiac diseases with phenotype of Naxos disease variant. Pediatric patients who demonstrated dilated cardiomyopathy but without skin and hair manifestations were excluded from our study.

### Study parameters

Data collected from the patients included demographic data, family history, cutaneous and cardiac phenotypes, ECG and echocardiography results, genetics analysis findings, and major cardiac events (such as arrhythmias, hospitalization, ICD implantation, heart transplant, or death) observed during the proband's follow-up. All information was gathered from patient medical records.

### Tests

*Cardiology Examination:* Echocardiographic parameters were analyzed, including the assessment of left ventricular systolic function using left ventricular ejection fraction (LV-EF) measured by M-mode and the four-chamber Simpson’s method, as well as left ventricular global longitudinal strain (LV-GLS). Right ventricular systolic function was evaluated using fractional area change (RV-FAC). Additionally, left ventricular end-systolic and end-diastolic dimensions (LVESD/LVEDD) were assessed.

All echocardiographic parameters were obtained from the first echocardiographic studies performed at the time of the initial assessment at our center. Arrhythmia events were analyzed using electrocardiography (ECG) and 24-h Holter monitoring.

*Genetic testing:* Whole blood samples were collected from patients and most first-degree relatives following informed consent. Genomic DNA was extracted using the Puregene Blood Core Kit C (Qiagen Inc., Valencia, CA, USA, REF 158389). Whole exome sequencing (WES) was performed on index cases using the Illumina HiSeq2000 platform, achieving an average coverage of ~ 100x, with approximately 97% of the targeted bases covered at > 10x. Reads were mapped to the genome build (hg19/b37). Sample preparation and enrichment were conducted using Agilent's SureSelect protocols.

Sequence reads were aligned to the human genome reference (hg19/b37) using the Burrows-Wheeler Aligner package, version 0.6.2. Disease-causing variants reported in ClinVar and/or the Human Gene Mutation Database (HGMD) were prioritized. Variants with a minor allele frequency (MAF) < 0.01 in the gnomAD database, specifically within genes associated with cardiomyopathies (CMP), were initially considered. Segregation analysis of the identified variants was conducted using all available samples from first-degree relatives of the index cases.

### Statistical analysis

Descriptive data were summarized using descriptive statistics. Statistical analyses were performed using the SPSS software package. Continuous variables were presented as mean ± standard deviation, with minimum and maximum values primarily reported for echocardiographic parameters. Categorical variables were expressed as percentages.

### Ethical consideration

The study was conducted in accordance with the principles of the Declaration of Helsinki (2013), the ICH Harmonized Tripartite Good Clinical Practice Guidelines, the policies and guidelines of the Research Advisory Council (RAC), and the laws of Saudi Arabia (RAC #2,211,178).

As this was an observational study without direct patient management, a waiver of signed consent was obtained from the Research Ethics Committee (REC). Patients were recruited from the Pediatric Heart Failure outpatient clinic. Data were recorded on a data collection sheet and subsequently stored electronically. All data were handled confidentially and anonymized to ensure patient privacy.

## Results

**Fig. 1:**
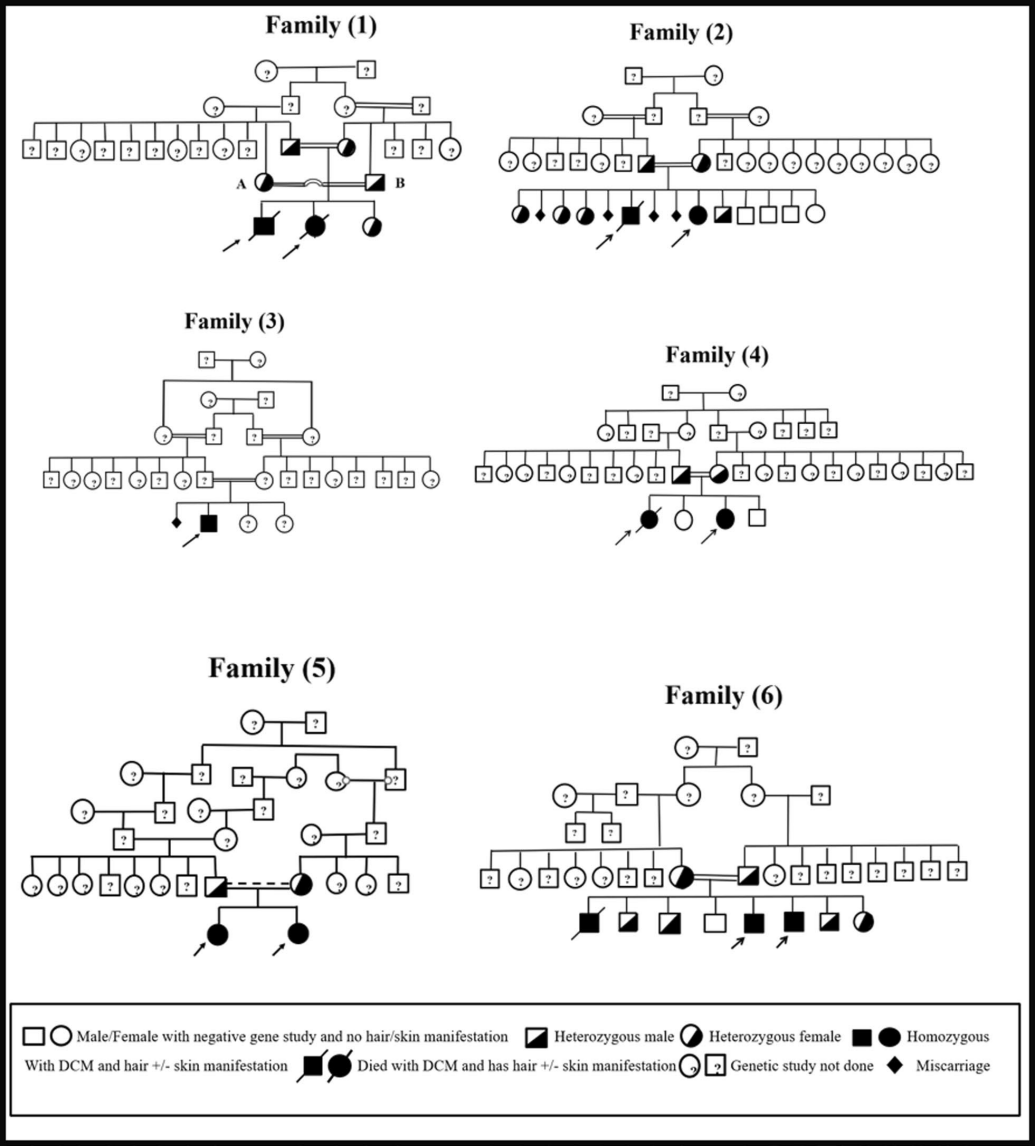
Family Pedigrees

We report ten cases from six Saudi families presenting with an autosomal recessive cardio-cutaneous disorder, identified as a Naxos disease variant (Carvajal syndrome). The cohort included four males and six females, with all families having a history of consanguinity, as illustrated in Fig. 1. The mean age at diagnosis was six years. All patients (100%) exhibited woolly hair, while five (50%) also presented with palmoplantar keratoderma (Table 1).

**Table 1:** Clinical data

Family No	Case No	Age At Dx (Year)	Sex M: male, F: Female	Family History	Cutaneous Phenotype	
					**Wooly Hair**	**Keratoderma**
1	1	4	F	+	+	−
	2	4	M	+	+	−
2	3	8	F	+	+	+
3	4	6	M	−	+	+
4	5	3	F	+	+	+
	6	7	F	+	+	+
5	7	7	F	+	+	−
	8	5.5	F	+	+	+
6	9	11	M	+	+	−
	10	5	M	+	+	−
	10 cases	Mean age: 6 yr Range:3 − 11yr	M: F 4:6	90%	100%	50%

The patients were referred from their local hospitals to our advanced heart failure clinic with a diagnosis of severe left ventricular cardiomyopathy for comprehensive evaluation and potential heart transplant work-up. Most patients were initially seen at their local hospitals with symptoms such as shortness of breath, abdominal pain, and vomiting. Chest X-rays revealed cardiomegaly, prompting further assessment with echocardiography. At the local hospitals, the patients were managed with anti-heart failure medications in accordance with pediatric heart failure management guidelines. Patient No. 8 was evaluated due to a positive family history and was found to have depressed left ventricular systolic function, leading to early diagnosis.

Upon the first evaluation at our center, all patients exhibited severely dilated systolic, diastolic, and left ventricular dimensions with significantly elevated Z-scores (LVESD Z-score mean: + 9.4; LVEDD Z-score mean: + 5.2). Left ventricular systolic function was markedly impaired, with a mean LV-EF of 24.1% assessed by both M-mode and the four-chamber Simpson’s method, and a mean LV-GLS of –7.3% (Table 2).

**Table 2:** Echocardiographic parameters at diagnosis

Variable	Mean	Std Dev	Minimum	Maximum	Interquartile Range (IQR)	Median
LV-EF by M-mode (%)	25.7	11.5	10	45		24
LV-EF by Simpson's (%)	24.1	9.4	11	41		21
LV-GLS (%)	− 7.3	3.7	− 3.5	− 13		− 6
LVESD (Z-score)	+ 9.4	−	+ 4.8	+ 14.3		+ 9.7
LVEDD (Z-score)	+ 5.2	−	+ 3.1	+ 7.2		5.7
RV-FAC (%)	21	−	13	38		20

LV-EF: left ventricular ejection fraction. LV-GLS: left ventricular global longitudinal stain. LVESD: left ventricular end-systolic dimension by Z-score using Xcelera Classic Z-score method. LVEDD: left ventricular end-diastolic dimension by Z-score using Xcelera Classic Z-score method. RV-FAC: right ventricular fractional area change

Right ventricular systolic function was also compromised in nine cases (90%), with a mean RV-FAC of 21%. The exception was Patient No. 8, who presented with normal right ventricular systolic function (RV-FAC: 38%) at the time of initial evaluation at our center. However, subsequent echocardiographic assessments of this patient revealed a progressive decline in right ventricular systolic function, which eventually fell into the mild impairment range.

All cases (100%) exhibited sinus rhythm. Frequent isolated premature ventricular contractions (PVCs), predominantly with left bundle branch block (LBBB) morphology indicating a right ventricular origin, were observed in nine patients (90%) [Table 3]. Only one patient (Case No. 8) had no documented sustained ventricular arrhythmias and demonstrated normal right ventricular systolic function at presentation.

**Table 3:** Cardiac Events

Family No	Case No	Arrhy	Type Of Arrhy	ICD			Transplant			Death		
				** + / − **	Age (Yr)	Time Of Insertion From Dx (yr)	+ / −	Age (Yrs)	Yrs From Dx	+ / −	Age (Yrs)	Yrs From Dx
1	1	+	Isolated PVCs & sustained PVCs (3%)	−	−	−	−	−	−	+	6	2
	2	+	Isolated PVCs (5%)	−	−	−	−	−	−	+	5	1
2	3	+	Isolated PVCs (2%)	−	−	−	+	10	2	−	−	
3	4	+	Sustained VT	+	10	4	+	10	4	−	−	
4	5	+	Isolated PVCs (3.5%)	−			−		−	+	4	1
	6	+	Isolated PVCs (4.2%)	−			−		−	−	−	
5	7	+	Isolated PVCs (5.2%)	−			+	8	1	−	−	
	8	−	−	−			−		−	−	−	
6	9	+	Isolated PVCs/ Sustained VT (4.7%)	+	12	1	−		−	−	−	
	10	+	Isolated PVCs & Sustained VT (2%)	+	14	2	+	19	7	−	−	
		9 (90%)	9 (90%)	3 (30%)	Mean: 12 yr	Mean: 2.3 yr	4 (40%)	Mean: 11.8 yr	Mean: 3.5 yr	3 (30%)	Mean: 5 yr	Mean: 1.3 yr

This table includes cardiac events: arrhythmias and type of arrhythmia, age of ICD implantation and time of implantation from the diagnosis time, heart transplant, if any, age of transplantation, and years of the implantation from the diagnosis time. The same thing applies to deaths. ICD: implantable cardioverter defibrillator. Arrhy: Arrhythmia. PVCs: premature ventricular contractions, VT: ventricular tachycardia. Dx: diagnosis. Yrs: years

Frequent, sustained ventricular tachycardia (VT) with LBBB morphology was reported in four patients (40%) (Cases 1, 4, 9, and 10). These episodes proved resistant to maximum anti-arrhythmic therapies, including beta-blockers, amiodarone, and Mexiletine. Case No. 4 experienced severe, uncontrolled VT despite intensive treatment with beta-blockers, amiodarone, and lidocaine infusion. VT was eventually controlled after the initiation of Mexiletine, allowing for a gradual tapering of lidocaine. This patient required urgent implantation of an implantable cardioverter defibrillator (ICD) due to severely decompensated heart failure while awaiting a heart transplant, which was successfully performed shortly thereafter. Notably, this patient presented with severely dilated and depressed biventricular systolic function (Table 2).

Additionally, two siblings from the same family (Cases 9 and 10, Family No. 6) survived cardiac arrest caused by malignant sustained ventricular arrhythmias. Emergency ICD implantation was performed for both, and one later underwent heart transplantation. Tragically, another sibling from this family, who exhibited the same hair phenotype as his affected siblings, experienced sudden cardiac death at a local hospital. A gene study was not conducted for this sibling, and the lack of referral to our center meant no clinical or paraclinical data were available, precluding inclusion in this study.

Additionally, three of the ten patients (30%) required ICD implantation for severe, sustained, and uncontrollable ventricular tachycardia that was refractory to maximum medical therapy (Patients No. 4, 9, and 10). The mean age at ICD implantation was 12 years, with a mean interval of 2.3 years from diagnosis to implantation. This highlights the rapid and severe progression of uncontrolled ventricular arrhythmias in Naxos and Naxos-like syndromes (Table 3).

All ten cases underwent evaluation for heart transplantation. Among them, four patients (40%) received heart transplants at a mean age of 11.8 years, with an average of 3.5 years from diagnosis to transplantation (Table 3). Tragically, three patients (30%) died suddenly at their local hospitals. Although detailed information on the causes of death is unavailable, arrhythmias are the most likely cause. Their deaths were discovered when they failed to attend follow-up appointments. The mean age at death was five years, with an average of 1.3 years from diagnosis to death (Table 3).

Three patients remain on the waiting list for heart transplantation. One of them (Patient No. 6) recently underwent implantation of a left ventricular assist device (LVAD, HeartMate 3) as a bridge to transplantation while awaiting a compatible donor. Unfortunately, cardiac MRI could not be performed for any of the patients due to their severely depressed left ventricular function at presentation, which made the use of general anesthesia for MRI unfeasible.

Regarding the genetic test results for our patients (Table 4), eight individuals were found to be homozygous for pathogenic variants in the Desmoplakin *(DSP)* gene. Notably, all variants were pathogenic, except for a de novo mutation identified in Family No. 6. Two patients (Patient No. 2 from Family No. 1 and Patient No. 5 from Family No. 4) exhibited identical phenotypic features to their siblings, including woolly hair and palmoplantar keratosis. Both were referred with severe decompensated cardiomyopathy but unfortunately passed away before genetic testing could be conducted.

**Table 4:** Genetic Study

Family No	Patient No	Gene	Result	Gene study for parents
1	1	DSP **NM_004415.4**	Homozygous for c.4297C > T (p.Gln1433Ter)	Father and mother both are Heterozygous for c.4297C > T; p.Gln1433Ter. mutation
	2	–	Unknown	
2	3	DSP	Homozygous for c.5242delA (p.Thr1748ProfsTer5)	Father and mother both are Heterozygous for c.5242delA (p.Thr1748ProfsTer5)
3	4	DSP *NM_004415.2*	Homozygous for c.4297C > T (p.Gln1433Ter)	The family has refused genetic screening for other members
4	5	-	Unknown	Father and mother both are Heterozygous for c.4999C > T (p.Gln1667Ter)
	6	DSP *NM_004415.2*	Homozygous for c.4999C > T (p.Gln1667Ter)	
5	7	DSP ***NM_004415.4***	Homozygous for c.4531C > T (p.Gln1511Ter)	Father and mother are both Heterozygous for For c.4531C > T (p.Gln1511Ter)
	8	DSP ***NM_004415.4***	Homozygous for c.4531C > T (p.Gln1511Ter)	
6	9	DSP ***NM_004415.2***	Homozygous c.1459A > G (p.Asn487Asp)	
	10	DSP ***NM_004415.2***	Homozygous c.1459A > G (p.Asn487Asp)	

**DSP:** Desmoplankin gene

In total, five families had a documented history of dilated cardiomyopathy, four of whom also reported a history of sudden cardiac death. One family (Family No. 3) had no confirmed history of either dilated cardiomyopathy or sudden cardiac death. This family also declined genetic testing for other members, limiting further analysis.

## Discussion

Naxos disease and its variants are rare autosomal recessive genetic disorders, clinically characterized by hyperkeratosis of the palms and soles, woolly hair, and cardiomyopathy [1, 10].

In 1986, Protonotarios et al. [1] reported nine cases (six males and three females) of palmoplantar keratosis from four families on the Greek island of Naxos. Pedigree analysis suggested that this condition was inherited as an autosomal recessive trait. Six patients presented with a history of palpitations and occasional syncope, with three experiencing severe VT with LBBB configuration, which was resistant to medical therapy in two cases. One patient who experienced PVCs died suddenly. Seven patients exhibited dilated right ventricular end-diastolic dimensions, and two also had left ventricular dilation with diffuse biventricular hypokinesia. The group hypothesized that their cases represented a familial autosomal recessive disorder characterized by palmoplantar keratosis and right ventricular dysplasia, which could lead to potentially fatal arrhythmias. Regarding the underlying genetic basis, a two-base-pair deletion in the plakoglobin (cell adhesion protein) gene (Pk2157del2TG) located on 17q21 was identified as the cause of Naxos disease [3, 11, 12]. This mutation was found in 13 families from Greece and one from Turkey [3, 5]. In the variant form of Naxos disease, two different mutations in the Desmoplakin gene, affecting the C-terminal of the protein (Dsp7901del1G and DspG2375R), were identified as causative in families from Ecuador and Israel (Arab families) [3, 13, 14].

In 2004, Bukhari et al. [4] reported a two-year-old Saudi girl with diffuse palmoplantar keratosis and woolly hair. Her 30-year-old aunt displayed the same skin and hair phenotype, but neither of them had any cardiac issues. The authors highlighted these cases as an early indication of Naxos disease, with the potential for cardiomyopathy to develop in the future, emphasizing the need for early and vigilant follow-up. Genetic studies were conducted for both the two-year-old girl and her aunt, though the results were still pending at the time of publication.

Later in 2004, Stuhrmann, Bukhari, and their colleagues [9] reported the absence of the Pk2157del2 frameshift mutation in the same two-year-old girl and her 30-year-old aunt. This led them to conclude that Naxos disease in this Saudi Arabian family was not caused by the same mutation identified in Greek families, providing evidence against the involvement of the plakoglobin gene. However, the focus of their study remained on plakoglobin, as it was the gene previously implicated in the Greek families from Naxos Island, where the disease was originally described. Notably, the Desmoplakin gene was not included in their genetic analysis of these patients.

Mandal et al. [15] reported two Saudi siblings with Carvajal syndrome, a variant of Naxos disease. The 10-year-old girl and her 12-year-old brother were admitted to the pediatric intensive care unit (PICU) with manifestations of decompensated heart failure. Both exhibited palmoplantar keratosis and woolly hair and were found to have dilated right and left ventricles on echocardiography, along with depressed left ventricular systolic function. Unfortunately, both siblings passed away shortly after admission due to severe heart failure. Genetic analysis, using polymerase chain reaction (PCR), revealed a missense mutation in the *DSP* gene, confirming the diagnosis of Naxos disease variant (Carvajal syndrome) in both patients.

In our study, we reported ten patients with palmoplantar keratosis, half of whom also had woolly hair, which is a characteristic feature commonly associated with the Naxos disease variant. Most of our patients exhibited left ventricular cardiomyopathy, a hallmark of the condition. Genetic testing revealed that they were homozygous for mutations in the *DSP* gene, with all variants located in the same protein domain, except for one family (Family No.6, Table 4), where the mutation was located in the SH3 domain. For couples who were identified as carriers of the DSP gene, we offered pre-implantation genetic diagnosis (PGD) and provided comprehensive counseling on Naxos disease, its inheritance patterns, and the role of consanguinity in increasing the risk of autosomal recessive disorders. In Family No.1 (Fig. 1), when genetic testing revealed that both potential marriage partners were heterozygous for the DSP gene, they decided against marrying each other, in order to prevent passing on the genetic condition.

**Fig. 2:**
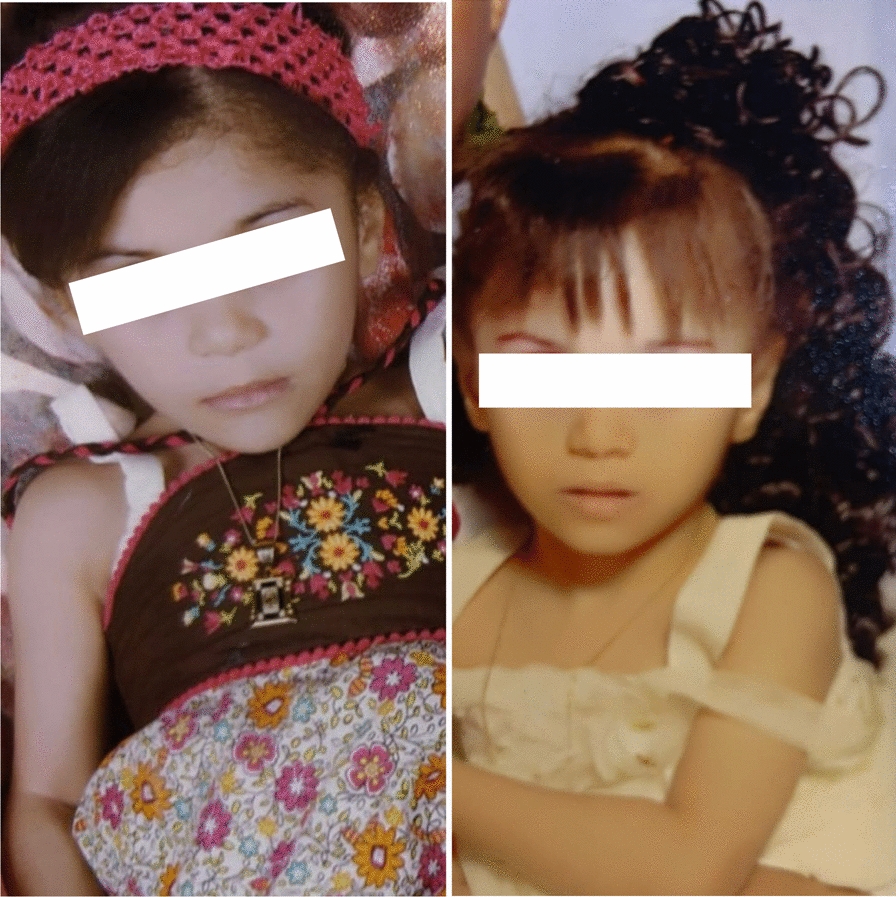
Patient Number 7 and 8 from Family 5

All patients presented with significantly dilated left ventricular systolic and diastolic dimensions, accompanied by severely impaired left ventricular systolic function. Additionally, nine patients exhibited right ventricular dysfunction. Ventricular arrhythmias, both sustained and non-sustained, were reported in nine of the patients. Of these, three required ICD implantation, four underwent heart transplantation, and three unfortunately passed away. To bridge the waiting period for a heart transplant, one patient recently underwent implantation of a left ventricular assist device (LVAD, HeartMate3) (Fig. 2).

## Conclusion

Woolly hair and keratosis of the extremities are hallmark early signs of Naxos disease and its variants, which are associated with rapidly progressing cardiomyopathy and arrhythmias. These hair and skin abnormalities serve as reliable indicators of the potential onset of cardiomyopathy. While mutations in the DSP gene are primarily linked to left ventricular dysfunction, mutations in the plakoglobin gene predominantly affect the right ventricle.

Naxos disease and its variants carry a high risk of arrhythmias and sudden cardiac death. Therefore, patients with palmoplantar keratosis and woolly hair should promptly be referred to a pediatric cardiology team for comprehensive cardiac and genetic evaluation. Early and continuous monitoring is essential to detect cardiomyopathy at its onset, enabling timely heart failure management, heart transplantation evaluation, and initiation of antiarrhythmic therapies. Properly timed ICD implantation is crucial to prevent sudden cardiac death in these high-risk patients. Furthermore, genetic testing for family members is critical to identify gene carriers, with thorough counseling being essential, especially in Arab populations where consanguineous marriages are prevalent.

### Study limitations

Our study has a couple of limitations, including the small sample size (only 10 cases) and its retrospective descriptive nature, focusing on patients already diagnosed with severe dilated cardiomyopathy. Another limitation is the lack of baseline echocardiographic assessments to evaluate right and left ventricular function at the initial presentation, which makes it difficult to determine whether right or left ventricular dysfunction developed first. Additionally, the exact cause of death in the local hospital remains unclear. However, we hypothesize that arrhythmias were the cause, given the sudden nature of the deaths, and we reached out to the families to verify medication compliance. Furthermore, histological analysis of palmoplantar keratosis was not performed for our patients.

## Data Availability

The data is unavailable due to privacy and ethical restrictions.
